# Injection of a Soluble Fragment of Neural Agrin (NT-1654) Considerably Improves the Muscle Pathology Caused by the Disassembly of the Neuromuscular Junction

**DOI:** 10.1371/journal.pone.0088739

**Published:** 2014-02-10

**Authors:** Stefan Hettwer, Shuo Lin, Stefan Kucsera, Monika Haubitz, Filippo Oliveri, Ruggero G. Fariello, Markus A. Ruegg, Jan W. Vrijbloed

**Affiliations:** 1 Neurotune AG, Schlieren, Zurich, Switzerland; 2 Biozentrum, University of Basel, Basel, Switzerland; Georgia Regents University, United States of America

## Abstract

Treatment of neuromuscular diseases is still an unsolved problem. Evidence over the last years strongly indicates the involvement of malformation and dysfunction of neuromuscular junctions in the development of such medical conditions. Stabilization of NMJs thus seems to be a promising approach to attenuate the disease progression of muscle wasting diseases. An important pathway for the formation and maintenance of NMJs is the agrin/Lrp4/MuSK pathway. Here we demonstrate that the agrin biologic NT-1654 is capable of activating the agrin/Lrp4/MuSK system *in vivo*, leading to an almost full reversal of the sarcopenia-like phenotype in neurotrypsin-overexpressing (SARCO) mice. We also show that injection of NT-1654 accelerates muscle re-innervation after nerve crush. This report demonstrates that a systemically administered agrin fragment has the potential to counteract the symptoms of neuromuscular disorders.

## Introduction

Neuromuscular disorders are a group of mostly mono-genetic diseases that affect the motor system. The cells involved can be motor neurons, peripheral nerves or the muscle and they include diseases such as spinal muscular atrophy (SMA), amyotrophic lateral sclerosis (ALS), Charcot Marie Tooth (CMT) syndrome, congenital myasthenic syndrome (CMS) or muscular dystrophies. Several lines of evidence indicate that the malfunctioning of the neuromuscular junction (NMJ) contributes to most if not all of those diseases [1], [2]. Besides these rather rare diseases, enhanced muscle loss during ageing, called sarcopenia, has been shown to also involve the disassembly of the NMJ [3]–[5]. Thus, a key element of counteracting muscle atrophy might be the maintenance of NMJs.

The formation and maintenance of NMJs is driven by the agrin/Lrp4/MuSK pathway [6]. During development, the extracellular matrix molecule agrin is released from the innervating motor neuron to bind to its receptor Lrp4, which in turn activates the receptor tyrosine kinase MuSK. The local activation of MuSK, via the binding to the cytosolic adaptor molecule Dok-7, then causes the formation of the postsynaptic apparatus at the site of nerve-muscle contact [7], [8]. NMJs start as small, pre-formed plaques and mature from perforated plaques into final, fully innervated pretzel-like structures [7]. One of the hallmarks of those postsynaptic structures is the high concentration of acetylcholine receptors (AChRs), which reaches a density of more than 10,000 molecules/µm^2^ [9]. The function of agrin to assemble the postsynapse can be reproduced in tissue culture as overnight incubation of aneural myotubes with recombinant neural agrin induces high-density AChR clusters [10]. The use of those assays has shown that a short amino acid insert at the C-terminal end of agrin (called z insert) is essential for this activity. The agrin gene codes for a family of basal lamina proteins that differ in function and distribution [11]. Splice site specific knock-out experiments in mice have confirmed that the z-insert is indeed required for NMJs to form *in vivo* [11], [12].

The agrin/Lrp4/MuSK signaling pathway is essential for survival as mice deficient for either agrin, Lrp4, MuSK or Dok-7 die at birth because of respiratory failure [6], [13]–[17]. Moreover, mutations or auto-antibodies against the elements of the agrin/Lrp4/MuSK pathway lead to myasthenia [18], [19]. Thus, there is compelling evidence that the agrin/Lrp4/MuSK pathway is also essential for the maintenance of the NMJ suggesting that manipulation of this pathway may be a promising way to ameliorate the phenotype of neuromuscular disorders. Indeed, genetic elevation of MuSK signaling has recently been shown to improve motor performance and to delay denervation in a mouse model for ALS [20].

Recently, neurotrypsin has been identified to affect the agrin/Lrp4/MuSK pathway. Neurotrypsin is a synaptic serine peptidase that cleaves agrin at two specific sites and thus inactivates its AChR clustering activity [21]. The cleavage products, C-terminal agrin fragments (CAFs), are released into the circulation. Interestingly, CAFs are elevated in a subset of sarcopenia patients [22], suggesting that degradation of agrin at NMJs may contribute to sarcopenia. Indeed, overexpression of neurotrypsin in motor neurons in mice causes a sarcopenia-like phenotype characterized by muscle weakness, fragmentation of NMJs, higher serum levels of CAF and pathological abnormalities in the muscle, suggesting that it can serve as a mouse model for precocious sarcopenia [23]. The mice are thus called SARCO mice. The phenotype of SARCO mice can be fully reversed by overexpression of a neurotrypsin-resistant version of agrin, suggesting that replenishment of agrin might be an option in the treatment of sarcopenia.

In this study, we engineered a C-terminal fragment of mouse agrin, termed NT-1654, which is neurotrypsin-resistant, highly soluble and retains the AChR clustering activity. Here, we show that this recombinant agrin, administrated by subcutaneous injection, can stabilize the NMJs of SARCO mice and thus ameliorate the phenotype. We also provide evidence that re-innervation after sciatic nerve crush can be accelerated by NT-1654.

## Results

### NT-1654 is protected from neurotrypsin cleavage and is highly soluble

NT-1654 represents a 44 kDa fragment of the C-terminal end of murine agrin. It contains the 8 amino acid insertion at the z-site rendering it active for AChR clustering. To obtain a highly soluble fragment, the y site did not contain the 4 amino acid heparin binding site preventing the molecule to stick to the extracellular matrix. With this modification, the protein was soluble to concentrations of at least 100 mg/ml. To protect NT-1654 from cleavage by neurotrypsin at the β-site **(Fig. S1A)**, the lysine at position P1 was mutated to alanine as described in [21]. Size exclusion chromatography confirmed that NT-1654 is monomeric **(Fig. S1B)**. The protein could be purified to a high degree of purity as judged by size exclusion chromatography and sypro ruby staining on an SDS-PAGE gel **(Fig. S1C)**.

### NT-1654 shows AChR clustering activity *in vitro* and *in vivo*

**Figure 1:**
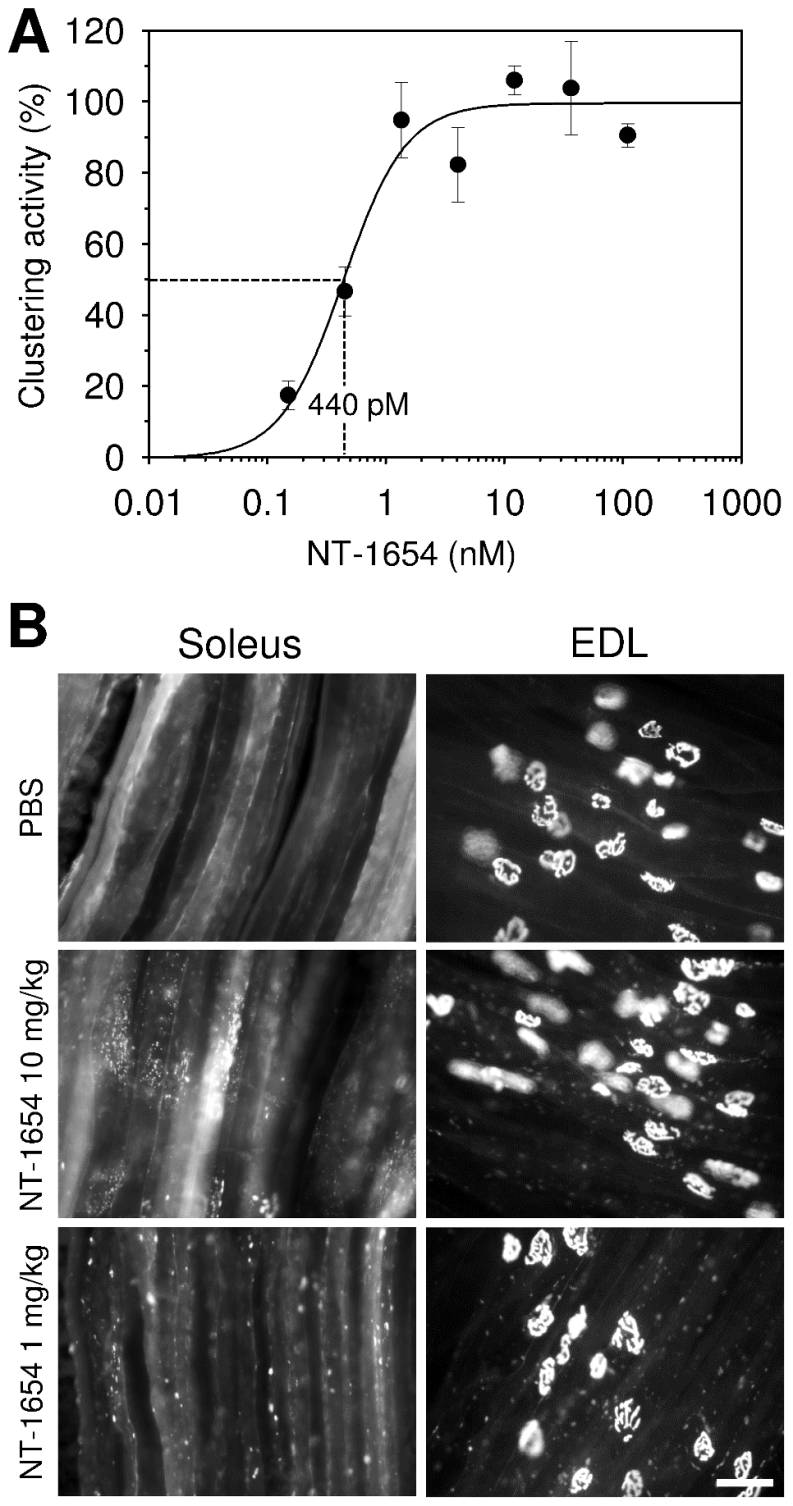
NT-1654 induces ectopic AChR clusters. **A:** Dose response curve of AChR-aggregating activity of NT-1654 *in vitro*. NT 1654 was applied on C2C12 myotubes and induced AChR clusters at the concentrations indicated in Fig. S2A. The area of AChR clusters per myotube was measured and normalized to the area of the myotubes. Percentage clustering activity was calculated by the mean value of 108, 36 and 12 nM to 100%. Data points represent mean ± standard error of triplicate cultures with 10 myotube segments counted in each. The curve was fitted to the data points with a sigmoidal regression. The EC_50_ was determined from the fit as 440 pM and indicated by the dotted lines. **B:** NT-1654 induces ectopic AChR clustering *in vivo*. NT-1654 induced ectopic AChR clusters in 6-days denervated *soleus* and EDL muscles. The ectopic AChR clusters were mainly present in the distal ends of *soleus* muscle whereas they were mainly at the NMJ zone of EDL muscle. No or only few clusters were observed upon injection of PBS. There is no obvious difference between 10 mg/kg and 1 mg/kg groups**.** Scale bar: 50 µm.

The AChR clustering activity was tested *in vitro* in differentiated mouse C2C12 myotubes. Upon administration of NT-1654 for 16 hours, AChRs formed aggregates on the myotubes in a dose-dependent manner **(Fig S2A).** The EC_50_ of NT-1654 was 440 ± 130 pM **(** **Fig. 1A** **)**. This value is in the range of the activity reported for the corresponding fragment (but containing the heparin binding site) derived from chick agrin when tested on primary chick myotubes [10]. A binding to full-length Lrp4 expressed in HEK293 cells could be demonstrated **(Fig. S2 B-D)**. To test whether NT-1654 would also induce AChR aggregates *in vivo*, we next injected it subcutaneously for 6 consecutive days into C57/Bl6 mice. To increase the expression of AChRs in the muscle, one hind leg was denervated by cutting the sciatic nerve. Mice were injected with PBS (Control), 10 mg/kg NT-1654 or 1 mg/kg NT-1654, starting immediately after denervation. *Soleus* and *extensor digitorum longus* (EDL) muscles were stained with Alexa555-labeled α-bungarotoxin to identify non-synaptic (ectopic) AChR clusters induced by NT-1654. Examination of the innervated leg showed that no or only few non-synaptic AChR clusters were induced by NT-1654. In contrast, NT-1654 induced numerous ectopic AChR clusters in the *soleus* and the EDL muscle of the denervated leg, while no or only few clusters were observed upon injection of PBS (**Fig. 1B**). There was no difference between the two dosage groups. The ectopic AChR clusters were found mainly at both distal ends of the *soleus* muscle whereas they appeared near the endplate band in the EDL muscle. These experiments show that NT-1654 has the expected AChR aggregation activity both *in vitro* and *in vivo*. The potency of NT-1654 in the AChR aggregation assay is very similar to that reported for the corresponding fragment of chick agrin supporting the conclusion that NT-1654 acts via activating the agrin/Lrp4/MuSK signalling pathway.

### NT-1654 ameliorates the weight and strength loss of SARCO mice

**Figure 2:**
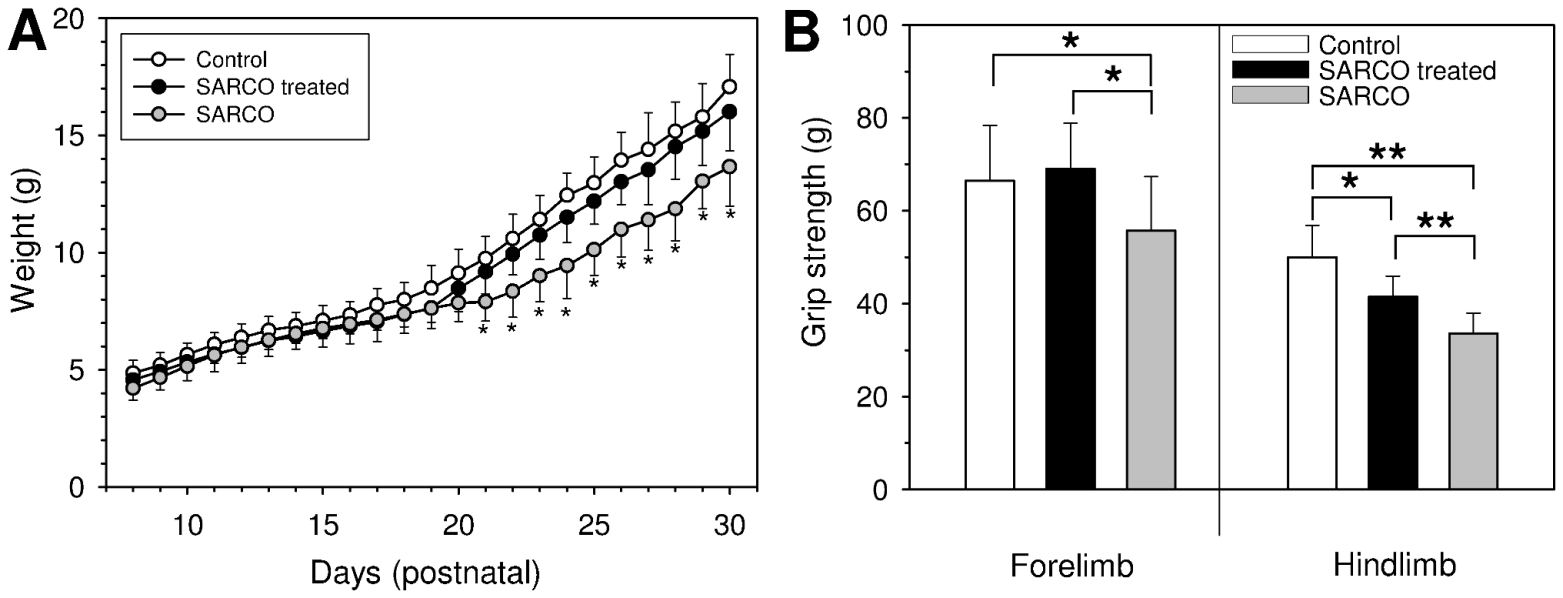
Weight chart and grip strength analysis of NT-1654 and vehicle treated SARCO mice. **A:** Weight recording of wild type littermates (Control), SARCO mice treated with NT-1654 (SARCO treated) and vehicle treated SARCO mice (SARCO). Treatment started at P8 s.c. with 10 mg/kg per day and ended at P30. Weight was recorded every day before the next dose was applied. For control, littermates (SARCO and Control) were simultaneously injected with PBS (vehicle). Starting at day 20, treated SARCO mice gain substantially more weight than the non-treated SARCO littermates. From day 21 on, the difference in weight became significant (*, p<0.05). Number of animals: wt  =  11; SARCO treated  =  9, SARCO  =  6. **B:** Grip strength measurements of Controls, SARCO mice treated with NT-1654 and SARCO mice at P30, the last day of compound administration. Bars represent mean values ± standard deviation. Treated SARCO mice showed significantly increased grip strength compared to the non-treated SARCO littermates. The forelimb strength reverted fully to Control level whereas the hindlimb strength was intermediate between wild type and SARCO mice. Number of animals: wt  =  11; SARCO treated  =  9, SARCO  =  6. * p<0.05; ** p<0.01.

To further test whether NT-1654 would be beneficial in situations where the NMJ disassembles, we tested its function to reverse the NMJ phenotype in SARCO mice. Because almost all NMJs of the animals are severely affected and partially denervated at postnatal day 8 (P8) [24], we started treatment at P8 and continued until P30. Mice were injected daily with 10 mg/kg NT-1654 s.c. (SARCO treated) or vehicle (PBS) alone (SARCO). The results were compared with vehicle-injected, non-transgenic littermates (Control). The weight of the mice was recorded every day during the treatment. At the beginning of the dosing, SARCO mice already had a small (0.5 gram) but significant weight deficit compared to the Controls (**Fig. 2A**). Starting around P20, SARCO mice significantly gained less weight compared to Controls. At P30, the average weight of SARCO mice was only 80% of Controls. In contrast, NT-1654 treated SARCO mice gained significantly more weight than vehicle-injected SARCO mice starting at P20. From that day on, the weight gain curve was parallel to the curve of Control animals and at P30, NT-1654 treated SARCO mice reached 94% of the weight of Controls.

At the last treatment day, grip strength of the fore limbs and hind limbs was recorded. SARCO mice had significantly reduced grip strength in their fore- and hindlimbs compared to Controls (80% and 61%, respectively; **Fig. 2B**). In contrast, the fore limb grip strength of NT-1654 treated SARCO mice was indistinguishable from Controls (104%) and the hind limb grip strength reached 80% of Control levels, which is an almost 20% enhancement compared to non-treated SARCO mice. These experiments thus show that NT-1654 can significantly preserve muscle force compared to the vehicle-treated SARCO mice.

### NT-1654 restores NMJ structures in SARCO mice

**Figure 3:**
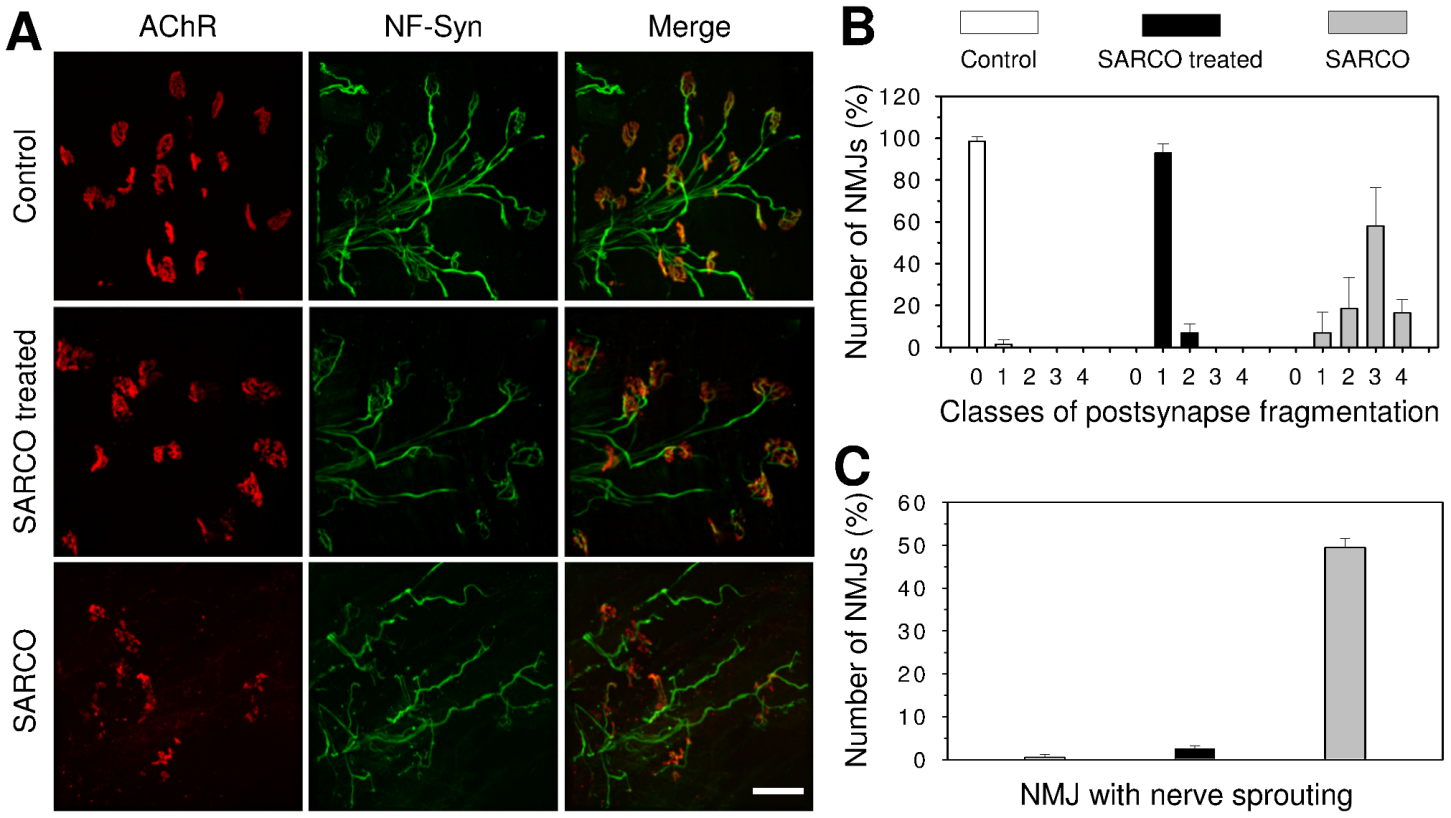
NMJ morphology of *m. soleus* of NT-1654 and vehicle treated SARCO mice. **A:** Confocal images of the NMJs in *soleus* muscle of P30 Control or SARCO mice, treated or not treated with NT-1654 as indicated. The postsynaptic AChRs were stained with Alexa-555 conjugate α-bungarotoxin (red) and the presynapse was stained with anti-neurofilament and synaptophysin antibodies (NF-Syn; green). The postsynapses were highly fragmented, partially or completely lost in *soleus* muscle of SARCO mice. The postsynapses of NT-1654 treated SARCO mice resembled those in Control mice, showing pretzel like structures and much less fragmentation. Scale bar: 50 µm. **B:** The postsynapses in Control mice showed fragmentation class 0 (no fragmentation, for illustration see figure S3). The postsynapses of NT-1654 treated SARCO mice were mostly classified into class 1, whereas the postsynapse of SARCO mice showed class 1-4 with a peak at class 3. **C:** Many NMJs of SARCO mice have terminal nerve sprouting, which was dramatically reduced in the NT1654 treated SARCO mice. Data present mean ± standard deviation, n  =  2 mice, 100 NMJs were counted in each mouse.

To test whether NT-1654 has any effect on the fragmentation of the NMJ [23], we next examined their structure at the end of the treatment at P30. Whole mount preparations of *soleus* muscle were used in which presynaptic structures were labelled by antibodies to neurofilament and synaptophysin followed by Alexa-488-conjugated secondary antibodies. Postsynaptic AChRs were labelled with Alexa-555-conjugated α-bungarotoxin. As illustrated in **Fig 3A**, NMJs of Control mice showed normal innervation and the typical pretzel-like structure. NMJs of SARCO mice treated with NT-1654 were almost indistinguishable from Controls. Only a slight fragmentation within the synaptic gutter was observed. The pretzel-like structure was clearly formed and each endplate was properly innervated with little or no signs of denervation. In contrast, NMJs of vehicle-treated SARCO mice appeared strongly fragmented and many of them were only partially innervated. Some of the postsynapses were completely lost. In addition, terminal sprouting of the presynaptic nerve was often observed. Some ectopic clusters were detected as well in non-treated SARCO mice, indicative of denervation.

To analyse the NMJ morphology quantitatively, the postsynapse fragmentation was classified into five categories as illustrated in **Fig. S3**. NMJs in *soleus* muscle of SARCO mice showed fragmentation class 1–4 with the majority of the NMJs being in class 3, whereas those of SARCO mice treated with NT-1654 were mostly classified as class 1 **(** **Fig. 3B** **)**. Approximately half of the NMJs of SARCO mice showed terminal sprouting and this number was strongly reduced to only 2.5% when treated with NT-1654 **(** **Fig. 3C** **)**. These results indicate that NMJs are more stable in the NT-1654 treated SARCO mice.

Koelle staining, which detects the activity of acetylcholine esterase of the diaphragm was also used to analyse the NMJs. Diaphragms of control animals showed the typical endplate band in the central region of the muscle fibers (**Fig. S4**). Individual NMJs were strongly labelled and showed the classical pretzel-like appearance. In contrast, NMJs of vehicle-treated SARCO mice stained only weakly and appeared highly fragmented. As expected, the endplate band was broadened. In contrast, NMJs of SARCO mice that were treated with NT-1654 resembled wild-type NMJs. The endplate band of treated SARCO mice appeared to be broadened compared to the endplate band of Controls. The overall endplate staining was not as strong as in Controls but markedly improved compared to untreated SARCO mice. In addition, the shape of the NMJs was virtually identical to that of control NMJs with indications of perforations similar to Controls. This result resembles the observations in *soleus* muscle, suggesting that NT-1654 treatment of SARCO mice lead to postsynaptic structures similar to those detected in WT animals.

### Muscle fibers of NT-1654 treated SARCO mice resemble the wild-type phenotype

**Figure 4:**
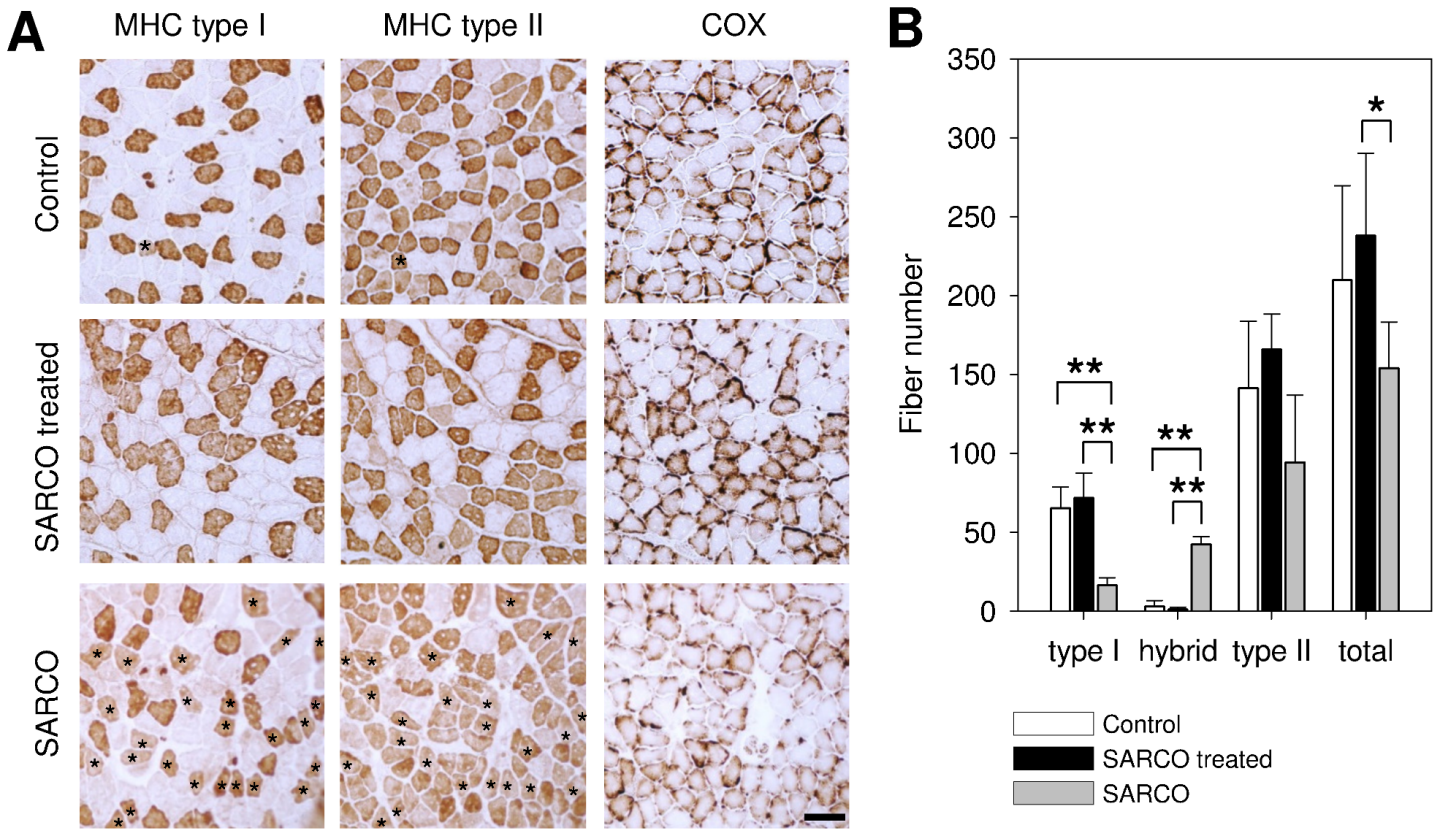
Fiber type distribution of *m. soleus* of NT-1654 and vehicle treated SARCO mice. **A:** Consecutive muscle cross sections of *soleus* stained with myosine heavy chain (MHC) specific antibodies or cytochrome C oxidase (COX) staining as indicated. Control and treated SARCO mice show clearly separated type I and II fibers. SARCO mice have a significantly increased amount of hybrid fibers (indicated with asterisks). Cytochrome C staining of *soleus* sections shows a massive reduction of reactivity in SARCO mice which is reverted back to WT levels in treated SARCO mice. **B:** Quantitative analysis of muscle fibers of *soleus.* Bars represent mean values ± standard deviation. Control and treated SARCO mice have almost no hybrid fibers and are indistinguishable from each other. Compared to treated ones, SARCO mice have significantly increased hybrid fibers. Type I fibers are significantly decreased. The total fiber number in SARCO mice is also significantly decreased compared to treated animals. Number of animals: wt  =  3; SARCO treated  =  5, SARCO  =  3. * p<0.05; ** p<0.01. Scale bar: 100 µm.

It has been previously described that the number of muscle fibers is reduced in SARCO mice compared to Controls [23]. At P30, we also observed a 27% loss of fiber number in the *soleus* muscles of SARCO mice. In contrast, the number of muscle fibers was as a trend even 14% higher than in Controls when SARCO mice were treated with NT-1654 (**Fig. 4B**).

During the course of the study it was also noted that SARCO mice contained many hybrid fibers. Such hybrid fibers are defined by the simultaneous expression of myosine heavy chains characteristic of type I and type II fibers (**Fig. 4A**). In *soleus* muscle of Control mice, hybrid fibers represented only 1% of all the fibers with remainder being 31% type I and 68 % type II fibers (**Fig. 4A, B**). SARCO mice showed a strong increase in hybrid fibers to 27.5% of the total fiber number (asterisks in **Fig. 4A**). Treatment of SARCO mice with NT-1654 normalized the fiber type distribution to Control levels (**Fig. 4**).

Mitochondrial changes and/or breakdown are key elements for specific pathways inducing muscle atrophy [25]. To assess whether there were also changes in mitochondrial function in SARCO mice, we also stained for cytochrome C oxidase (COX). SARCO mice displayed a massive reduction in COX staining, which was again normalized by the treatment of the mice with NT-1654 **(** **Fig. 4A** **)**. These results thus show that SARCO mice display many of the hallmarks of sarcopenia and that the treatment of the mice with NT-1654 is sufficient to normalize those hallmarks to Control levels.

### NMJ recovery after nerve crushing is enhanced by NT-1654

**Figure 5:**
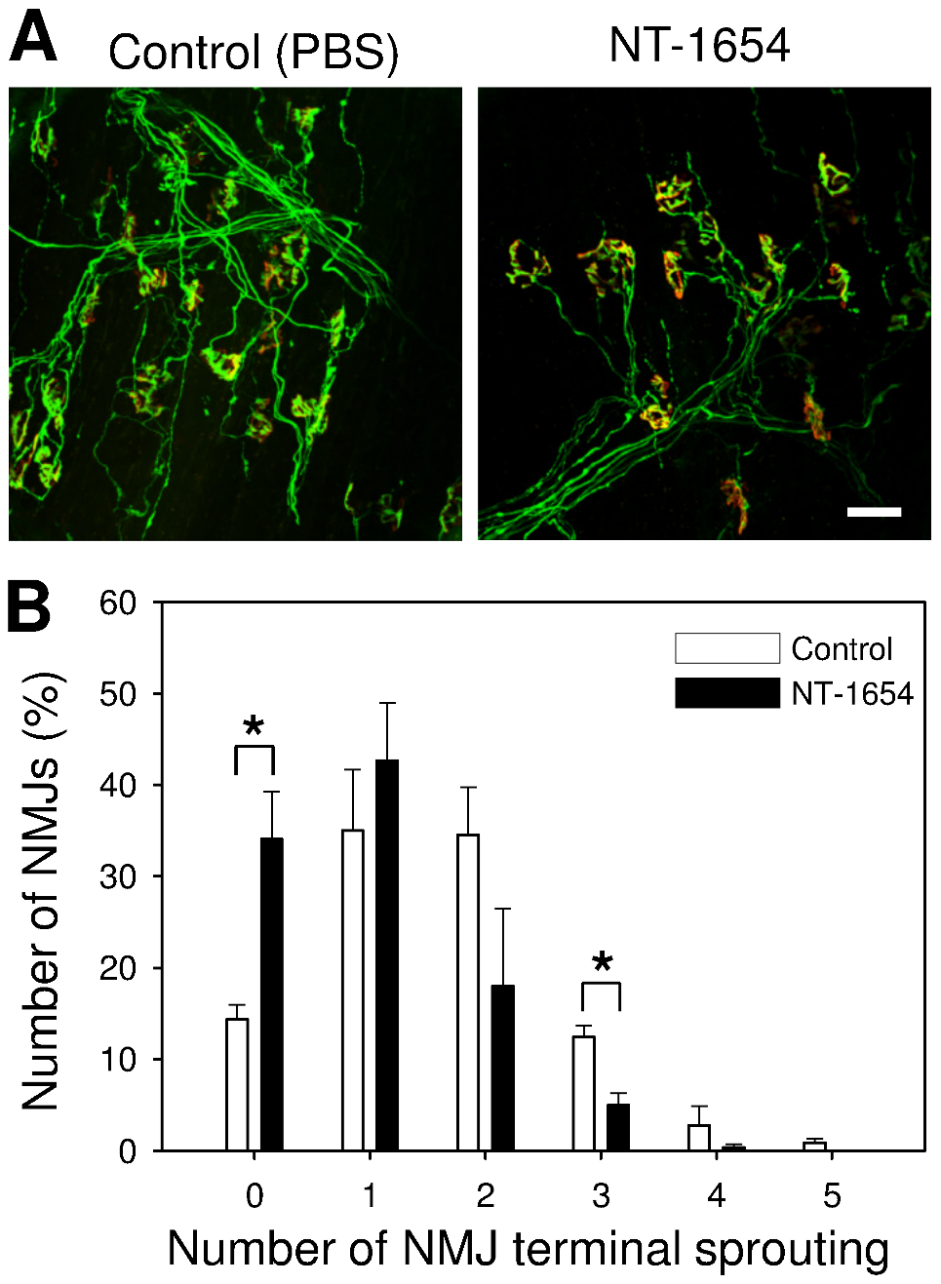
NMJ recovery after sciatic nerve crush. **A:** Confocal images of the neuromuscular junctions (NMJs) in *soleus* muscle of Thy1-YFP mice after 14 days sciatic nerve crushing, treated with NT-1654 or PBS (Control) as indicated. The postsynaptic AChR was stained with Alexa-555 conjugate α-bungarotoxin (red) and the presynapse was visualized by the transgenic expression of YFP in motor neurons (green). The NMJs in *soleus* muscle are fully re-innervated after 14 days sciatic nerve crushing. The NMJs of NT-1654 treated mice showed significantly less nerve sprouting than those treated with PBS (Control). The number of nerve sprouting in each NMJ was counted and shown in **B.** NMJs in the NT-1654 treated mice have significantly fewer events of nerve sprouting and significantly fewer number of nerve sprouts than those of Control mice. Data present mean ± standard error, *: p<0.05 (two ways t-test), 100 NMJs were counted in each mouse, n  =  3 mice, scale bar: 50 µm.

As NT-1654 restores the NMJs of SARCO mice from fragmentation and nerve sprouting, we next tested whether NT-1654 would also be beneficial for NMJ re-innervation. To establish the re-innervation model, the sciatic nerve of mice expressing YFP under the control of the Thy1 promoter [26] was crushed. The mice were treated either with PBS (Control) or with NT-1654 (10 mg/kg) by daily injection. Injection was started immediately after surgery and continued up to the analysis after 14 days. To examine the re-innervation of NMJs, the postsynaptic apparatus was stained with Alexa-555-conjugated α-bungarotoxin and the presynaptic nerve terminal was visualized by YFP expression in motor neurons. As shown in **Fig. 5A**, postsynaptic and presynaptic structures fully matched in *soleus* muscle in both groups, indicating that the muscle was fully re-innervated after 14 days. In control group, many NMJs were still multiply innervated and several of the nerve terminals sprouted, indicating that NMJs were still in the process of remodelling at this time point. Compared to Controls, NMJs of mice injected with NT-1654 showed little nerve sprouting **(** **Fig. 5A** **)**. Quantification revealed a significant lowering of the number of terminals with nerve sprouting upon NT-1654 injection compared to Control. While 86% of the NMJs from Controls showed terminal sprouting, this fraction was reduced to 66% in NT-1654 treated animals (**Fig. 5B**). Among those NMJs with nerve sprouting, the NT-1654-injected group also showed a significantly lower number of sprouts than the PBS-injected group. These results suggest that NT-1654 promotes directed nerve growth or accelerates the process of NMJ remodelling during re-innervation.

## Discussion

Optimal functioning of the NMJ depends on the formation of this highly complex structure at a specific site within the muscle fiber and, once this has been achieved, on its continuous maintenance. Both of these processes are governed by agrin through its interaction with the Lrp4/MuSK system [4]. In previous studies [23], [24] it was demonstrated that elevated levels of neurotrypsin produce deleterious effects on NMJs and muscles in SARCO mice. Since NMJ dysfunction is involved in the pathogenesis of many neuromuscular diseases [1], [2], we tested the hypothesis that a systemically delivered protein that retains the signalling function of neural agrin and, in addition, is resistant to cleavage by neurotrypsin may restore the proper communication between motor neurons and muscle fibers. Such an improvement of NMJ function was achieved by overexpression of a neurotrypsin-resistant full-length agrin in SARCO mice [23]. In that study, muscle fiber pathologies and loss of grip strength were restored to WT. Interestingly, this was not the case when WT agrin was overexpressed in the SARCO mouse background. This shows the importance of protecting the β-cleavage site of agrin from the deleterious cleavage by neurotrypsin.

The biologic NT-1654 is a 44-kDa protein derived from the C-terminal portion of agrin, which was engineered with the aim of retaining the agonistic activity on the Lrp4/MuSK receptor complex. This fragment does not contain any heparan sulphate chains or any binding sites for laminin, heparin or integrin. These structural binding elements were removed from the agrin molecule in order to optimize NT-1654 for tissue penetration without losing signalling activity. In addition, a site-specific mutation at the β-cleavage site was introduced to prevent its breakdown by neurotrypsin. The resulting molecule is monomeric, highly soluble and stable and can be produced in quantities of up to 900 mg/L from transiently transfected CHO-S cells, which are all highly desirable pharmacological properties. We here show that NT-1654 binds to Lrp4 and induces AChR clusters in C2C12 myotube (**Fig. S2**). The EC_50_ in the AChR clustering assay is 440 pM (**Fig. 1A**), which is similar to the activity of the C45 fragment of chick agrin [10], indicating that it has the activity of agrin and the action is mediated by the agrin/Lrp4/MuSK signalling pathway. This is confirmed by the formation of ectopic AChR clusters observed in the denervated *soleus* and EDL muscles **(** **Fig. 1B** **)**. The interesting observation on the differential distribution of ectoptic AChR clusters in both muscles may be caused by a differential expression pattern of MuSK or another factor regulated by innervation, which was reported by Punga et al [27] and Lin et al [28]. While *soleus* is a ‘delayed synapsing’ (DeSyn) muscle, EDL is a ‘fast synapsing’ (FaSyn) muscle. The *in vivo* experiment alsoindicates that NT-1654 reaches muscles when it is injected subcutaneously.

We next tested whether NT-1654 is able to normalize the altered morphology of the NMJs and to restore the muscle function in SARCO mice. SARCO mice overexpress neurotrypsin in motor neurons starting around P0 [29]. At P4, 50% of the NMJs are partially disassembled and the nerve terminals start to sprout. At P8, the dissolution of virtually all NMJs was observed [24]. The alternation of NMJs is associated with muscle pathology and diminished strength and performance [23]. We thus treated the mice from P8 to P30. After 23 days of systematic administration of NT-1654, the NMJ morphology of *soleus* and diaphragm muscles of SARCO mice was strongly ameliorated, showing nearly normal pretzel-like structure (**Fig. 3** **, Fig. S4**). These results thus provide strong evidence that NT-1654 is capable of rebuilding the disassembled NMJs. As NT-1654 is active in the AChR clustering assay, its effect is likely mediated by agrin/Lrp4/MuSK signalling. The ability of NT-1654 to reverse the sarcopenia-like phenotype was also observed when examining the fiber type composition of the *soleus* muscle. SARCO mice had a profound increase of hybrid fibers to 27.5% of the total fiber number **(** **Fig 4** **)**.

Several lines of evidence indicate that hybrid fibers are indicative of denervation [30]. NT-1654 reversed the hybrid fibers to the normal segregation of type I and type II fibers when administered systemically to SARCO mice, suggesting that it is capable of enhancing innervation. In addition, we also observed that the loss of muscle fibers due to disintegrated NMJs was stopped and the energy metabolism was reverted back to normal as indicated by the COX staining. This finding correlates with the observations described by Powers at al., [25]: it is known that upon induced muscle atrophy in humans, e.g. by immobilization, mechanical ventilation or by denervation, mitochondrial physiologic changes and/or breakdown are key elements for specific pathways inducing fiber atrophy. A possible involvement of the agrin/Lrp4/MuSK pathway in the physiological changes of the mitochondria has already been shown by [31] demonstrating swollen and irregularly shaped mitochondria in humans suffering from MuSK-dependent myasthenia gravis. Interestingly, this phenotype can also be observed for the mitochondria of SARCO mice ([24], refer to Fig 1K).

The improvements due to treatment of SARCO mice with NT-1654 on biochemical and cellular level are also manifested in physiological data: besides gaining weight similar to WT littermates, NT-1654-treated animals showed improvement in the grip strength **(** **Fig. 2** **)**.

The beneficial effect of NT-1654 was confirmed in a SARCO mouse independent nerve re-innervation model in Thy1/YFP mice. 14 days after sciatic nerve crush, the NMJs of the NT-1654-treated animals showed significantly fewer nerve sprouts than those of the PBS-treated Control group. This suggests that the process of re-innervation including nerve growth or the remodelling process after re-innervation has been accelerated by NT-1654. It can be assumed that the induction of ectopic AChR clusters could be one driving factor for accelerated re-innervation. It has been shown by others [32] that the induction of ectopic AChRs by agrin on the cultured myotubes leads to an accelerated innervation when they are transplanted into mice.

The results also show that subcutaneously administered NT-1654 reaches the target *in vivo* and exerts a beneficial function in SARCO mice. This is the first report of a systemically treatment of a mouse model for neuromuscular disorders with an engineered agrin fragment. Further investigations will reveal whether NT-1654 has also a beneficial effect in other disease models. For example, one could expect a beneficial effect of NT-1654 in Agrn^nmf380^ mice, a model for congenital myasthenic syndrome [33]. A treatment of mouse models for SMA or ALS may have beneficial effects, although the primary cause of the disease cannot be targeted by NT-1654. A strengthening of the NMJ may lead to retrograde signalling that stabilizes the motor neuron. Straight in line with this suggestion is the finding that moderate overexpression of MuSK in the SOD1/G93A ALS mouse model delayed the disease onset and reduced the extent of muscle denervation and thereby improved motor function [20]. A preservation of the NMJ may prevent or delay the “dying back” of motor neurons in ALS as discussed in detail elsewhere [34]. Future experiments will reveal if NT-1654 can be of benefit in the treatment of human neuromuscular diseases that directly or indirectly affect the agrin/Lrp4/MuSK pathway. Whether age dependent sarcopenia might be treatable by this molecule remains to be shown in corresponding animal models, like the aged rat model [35]. As an overexpression of neurotrypsin-resistant agrin in neurotrypsin knock-out mice could not prevent signs of neuromuscular degeneration at old age, only the subgroup of 30–40% sarcopenia patients with neurotrypsin dependent sarcopenia [22] might be a target. An important feature of NT-1654 is that properly innervated muscle fibers are not affected as we could not observe non-synaptic AChR clusters in fully innervated muscle, indicating that partially denervated and fragmented NMJs are mainly targeted. Thus, an adverse effect on healthy muscle is not expected.

## Material and Methods

### Expression and purification of NT-1654

The C-terminal 44 kDa fragment of murine agrin (NT-1654) was expressed using the pEAK8 vector (Edge biosystems). The protein starts with L1519 (A2ASQ1 (AGRIN_MOUSE), UniProtKB/Swiss-Prot) and ends with the natural C-terminus of agrin. The fragment contains the z-8 amino acid insert and in addition the lysine to alanine mutation which renders the protein protected against site specific neurotrypsin cleavage at the β-cleavage site [36]. NT-1654 was transiently expressed in CHO-S cells in suspension culture (Evitria, Switzerland) to a level of up to 900 mg/L. For purification, NT-1654 was precipitated from the cell culture supernatant using 25% PEG 6000. The pellet was resolved in ¼ x PBS and subjected to hydroxyl-apatite chromatography. NT-1654 was eluted in a potassium phosphate gradient and the purification success was monitored by SDS-PAGE. Purity of the protein was assessed by staining 5 µg of protein with Sypro Ruby (Invitrogen) after SDS-PAGE followed by quantification with a Stella Imaging system (Raytest) using the AIDA quantification software. After purification, 54% of the protein could be recovered with a purity >97% in endotoxin-free quality (<0.25 EU/mg). Endotoxin levels were determined using the Limulus Amoebocyte Lysate PYROGENT Plus kit (Lonza). NT-1654 appeared as monomeric in size exclusion chromatography on a superdex 200 10/300 GL column (GE healthcare) using PBS as running buffer.

### Lrp4 binding assay

HEK293 cells, grown to 80% confluency in a 35 mm dish were transfected with 4 µg of an expression construct coding for Lrp4-myc (mouse full length Lrp4, cloned into pBluescript II KS+_myc plasmid from E15.5 C57/Bl6 mouse embryo). After 48 hours culture, cells were washed and incubated for 40 min at 37°C/5% CO_2_ with 1 nM of NT-1639, i.e. a human version of NT-1654, used for reasons of better detectability with the used antibody. The recombinant C21 fragment of chick agrin [37] was used as a positive control. The bound NT-1639 or C21 agrin were detected by rabbit anti-mouse agrin antibody #204 [38] at 1:1000. The expression of Lrp4 was detected by anti-Myc mAb at 1:1000 **(Fig S2B-D).**

### *In vitro* AChR clustering assay

150,000 C2C12 myoblasts were seeded on 35 mm tissue culture dishes in proliferation medium (DMEM with 4.5 g/l glucose, L-glutamine and sodium pyruvate; 10% Fetal bovine serum; penicillin/streptomycin). Upon cells reaching confluence, the medium was replaced with differentiation medium (DMEM low glucose, L-glutamine and sodium pyruvate; 2% horse serum; penicillin/streptomycin). After 2 to 3 days, myoblasts fused to form myotubes. NT-1654, diluted in differentiation medium, was subjected in different concentrations to the C2C12 myotubes for 16 hours. Myotubes were stained for AChRs by adding 500μl of (1 µg/ml) rhodamine conjugated α-bungarotoxin (Invitrogen) to the dish and incubation for 45-60min at 37°C. The dishes were washed 3 times with 1–2 ml MEM and fixed with 95% ice cold ethanol for 5min at –20°C. Ethanol was removed and evaporated for 3 minutes until the dish was dry. The dishes were mounted with Citifluor **(Fig S2A)**. The area of AChR clusters per myotube was measured and normalized to area of the myotubes. For the calculation of the EC_50_, the mean value of AChR cluster area at 108 nM, 36 nM and 12 nM was set to 100% since the maximal clustering activity was achieved at these concentrations.

### Animal experiments

All animal experiments were performed on C57/Bl6 mice or derivatives thereof in strict accordance with Swiss guidelines for proper conduct of animal experiments and were approved by the Veterinary Authorities (Kantonales Veterinäramt Basel, Kantonales Veterinäramt Zürich) of the Cantons Basel and Zurich (Permission numbers 2593 for Basel and 18/2008; 31/2011 for Zurich). All surgery was performed under ketamine (111 mg/kg)/Zylaxine (22 mg/kg) for regular anesthesia and phenobarbital for terminal anesthesia, and all efforts were made to minimize suffering. To avoid inflammatory reactions, only endotoxin free protein solutions were used in the experiments. Mice were kept in Blue-Line cages on “abedd” bedding (both Indulab). A maximum number of 5 mice were allowed per cage which was supplemented with tissues and hiding places. Mice had continuous access to dry pellets from Kliba Nafag and fresh drinking water. The night/day cycle was 12h/12h and experiments were done predominantely at times when mice were active.

### *In vivo* AChR clustering assay

Mice were anesthetized with ketamine/Zylaxine and the sciatic nerve of C57/Bl6 mice was cut on the left leg. The right leg was sham operated for use as control. The mice were treated with PBS (Control) or NT-1654 (10 mg/kg or 1 mg/kg) immediately after denervation (once per day, s.c.). Six days after denervation, mice were sacrificed and their muscles were harvested. *Soleus* and EDL muscles were stained with Alexa555-conjugated α-bungarotoxin (Invitrogen) to examine the formation of ectopic AChR clusters.

### Transgenic mouse lines

The SARCO mice (B6.C3-Tg(PRSS12) 491 Zbz) expressing transgenic human neurotrypsin under the Thy1.2 promoter, are previously described by [24] and [29]. Genotyping was done as described in [29]. A detailed investigation on this mouse line is given in [23] (refer to Nto1 mouse line).

In order to examine the re-innervation process in the nerve crushed muscle, the Thy1-YFP transgenic mice [39] were used. The Thy1-YFP mice were purchased from the Jackson Laboratory (Stock Number: 003709) and housed in the animal facility of Biozentrum by backcrossing with C57/Bl6 mice.

### *In vivo* compound administration

All mice were dosed with 1 mg/kg or 10 mg/kg NT-1654 s.c. once a day for the time indicated. PBS was used as vehicle and was administered to both SARCO mice and wild type littermates (Control).

### Grip strength in the limbs

Grip strength was measured using a metal grip bar connected to a tension spring balance (Columbus Instruments, Columbus OH). To measure the strength in the fore limbs, the mouse was held by the tail and allowed to grab the bar. To measure the strength in the hind limbs, the mouse was held by the tail and the fore limbs and then allowed to grab the bar with the hind limbs. The measured strength was the maximal force needed to pull the mouse gently away from the bar. In each trial, every mouse was tested 5 times. Trials were performed at P9, P10, P16, P19 and recording was done at P30.

### NMJ stainings

Harvested muscles at the end of the treatment were prepared for histopathological examination. Isolated *soleus* was injected with 2 µg/ml of Alexa555-conjugated α-bungarotoxin and incubated for 30 min at room temperature. Muscles were washed 3 times with PBS and fixed with 2% PFA in PBS for 10 min. After cutting the muscle into bundles, they were permeabilized with 1% Triton X-100 and incubated in blocking solution (5% horse serum and 0.2% Triton X-100 in PBS) for 2 hours at 4°C. The bundles were then incubated with rabbit anti-neurofilament 200 (Sigma N4142, 1∶10000) and rabbit anti-synaptophysin (DAKO A0010, 1∶200) in blocking solution over night at 4 °C. The bundles were washed with blocking solution at 4°C. Then, they were incubated with Alexa488 conjugated goat anti-rabbit/ antibodies for 2 hours at 4°C. The bundles were washed with blocking solution at 4°C and mounted with Citifluor. Acetylcholine-esterase staining was done with a modified Koelle reaction [40].

### Morphological analysis of NMJs

To analyse the NMJ morphology quantitatively, the stained NMJs were observed under the Leica DM 5000B fluorescence microscope with 63x objective and classified into 5 categories according to the class of postsynapse fragmentation **(Fig S3)**. 0: normal NMJ without fragmentation; 1: light fragmentation with pretzel like postsynapse; 2: intermediate fragmentation; 3: severe fragmentation, the pretzel like postsynapse could not be recognized; 4: the postsynapse largely or completely disappeared. The terminal sprouts of the NMJs were counted as well. 100 NMJs of each mouse were counted.

### Nerve crushing

The sciatic nerve of Thy1-YFP mice was crushed using a forceps under ketamine/Zylaxine anesthesia. The mice were treated with either PBS or NT-1654 (10 mg/kg) by daily injection per s.c. immediately after operation for 14 days. To examine the re-innervation of NMJs, postsynapse was stained with Alexa-555-conjugated α-bungarotoxin and presynapse was visualized by YFP expression in motor neurons. The stained muscle bundles were observed under the Leica DM 5000B fluorescence microscope with 63x objective. The number of the terminal sprouts of the NMJs was counted.

### Muscle fiber analysis

Mice were perfused under ketamine/Zylaxine anesthesia with ice-cold 0.9% NaCl. The calf muscles of the leg were quickly dissected as a package and frozen in isopentane cooled with liquid nitrogen. Sections were cut at 12 µm in a cryostat at –20°C, mounted on “superfrost plus” (Menzel) slides and stored at –20°C until utilization. Staining of type I fibers was done with biotinylated BA-D5 antibody, staining of type IIA fibers with SC-71 antibody as described in [41]. For the determination of mitochondrial function cytochrome C oxidase staining was used as described in [42].

## Supporting Information

Figure S1 **Domain structure and appearance of NT-1654. A:** Schematic drawing of full length agrin and NT-1654 (modified from [22]). NT-1654 consists of the LG2, EGF4 and LG3 domains of agrin. At the y-site, no additional amino acids are inserted while at the z-site 8 amino acids are inserted. The neurotrypsin β-cleavage site is mutated at the P1 residue by replacing the lysine by alanine. Neurotrypsin cleavage sites are indicated as α-cleavage site and β-cleavage site. Heparan sulphate glycan chain attachment sites are marked by lollipop chains, glycosylation sites by lollipops. The three splice sites x, A/y, B/z are indicated. NtA: N-terminal agrin domain; F: follistation domains; LE: laminin EGF-like domains; S/T: serine/threonine rich segments; SEA: sea urchin sperm protein, enterokinase, and agrin domain; EG: EGF-like domain; LG: laminin globular like domain; SS: signal sequence; TM: transmembrane segment. **B:** Size exclusion chromatography revealed that NT-1654 is monomeric. It elutes at the expected retention time. The elution profile of the Biorad 151-1901 gel filtration standard is depicted in red and the corresponding molecular weights of the standard proteins are indicated above the curve. **C:** The protein could be purified to a high degree of purity as judged by Sypro ruby staining on an SDS-PAGE gel. 2 ug of protein were loaded.
(TIF)

Figure S2 **Related to** **Figure 1:** **NT-1654 has AChR clustering activity and directly binds to Lrp4. A:** NT-1654 induces ectopic AChR clustering *in vitro*, related to Fig. 1A. C2C12 myotubes were subjected to various amounts of NT-1654 as indicated and stained with α-bungarotoxin to visualize clusters of acetylcholine receptors (AChRs). The number and area of AChR clusters were dose-dependent. The cluster area (percentage of myotube area) was then used to determine the EC_50_ (see Fig. 1A). Scale bar: 50 µm. **B:** HEK293 cells were transfected with Lrp4-myc and positive cells were detected by anti-myc antibodies (green). Cell nuclei were visualized by DAPI staining. **C:** HEK 293 cells were transfected with Lrp4-myc and NT-1639 (a human version of NT-1654) was added. NT-1639 that was bound to Lrp4 was detected by the 204 anti-agrin antibody (green). Cell nuclei were visualized by DAPI staining. **D:** As a positive Control, a C21 fragment of human agrin is shown to bind to Lrp4 (green). Scale bar: 20 µm.
(TIF)

Figure S3 **Related to** **Figure 3:** **Categorization of postsynapse fragmentation.** In order to quantify the severity of postsynapse fragmentation, we observed the NMJs under the Leica DM 5000B fluorescence microscope with 63x objective. The postsynaptic AChRs were stained with Alexa-555 conjugate α-bungarotoxin (red) and the presynapse was stained with anti-neurofilament and synaptophysin antibodies (NF-Syn; green). Class 0: normal NMJ without fragmentation; class 1: light fragmentation with pretzel like postsynapse; class 2: intermediate fragmentation; class 3: severe fragmentation, the pretzel like postsynapse could not be recognized; class 4: the postsynapse largely or completely disappeared. Scale bar: 50 µm.
(TIF)

Figure S4 **Acetycholine esterase activity on diaphragm of NT-1654 and vehicle treated SARCO mice.** Koelle-staining of diaphragm of P30 mice as indicated. Left panels: 40 x, right panels 200 x. The Koelle-staining visualizes the acetylcholine esterase activity in the NMJs. NMJs of Control mice are stained intense and are arranged in a distinct narrow endplate band. The shape of the NMJs is roundish and some perforations can be observed. NMJs of SARCO mice are faintly stained and fragmented. The endplate band is broader as in Controls, comparable to that in treated SARCO mice. Staining in treated SARCO mice is less intense but distinct. The shape of the NMJs is mostly roundish with some perforations at the edge. Scale bar given for 200 x magnitude: 50 µm.
(TIF)
