# Supplementary figures and images for: Injection of a Soluble Fragment of Neural Agrin (NT-1654) Considerably Improves the Muscle Pathology Caused by the Disassembly of the Neuromuscular Junction
Source: PLoS One. 2014 Feb 10;9(2):e88739. doi: 10.1371/journal.pone.0088739 (PMC3919806; doi:10.1371/journal.pone.0088739)

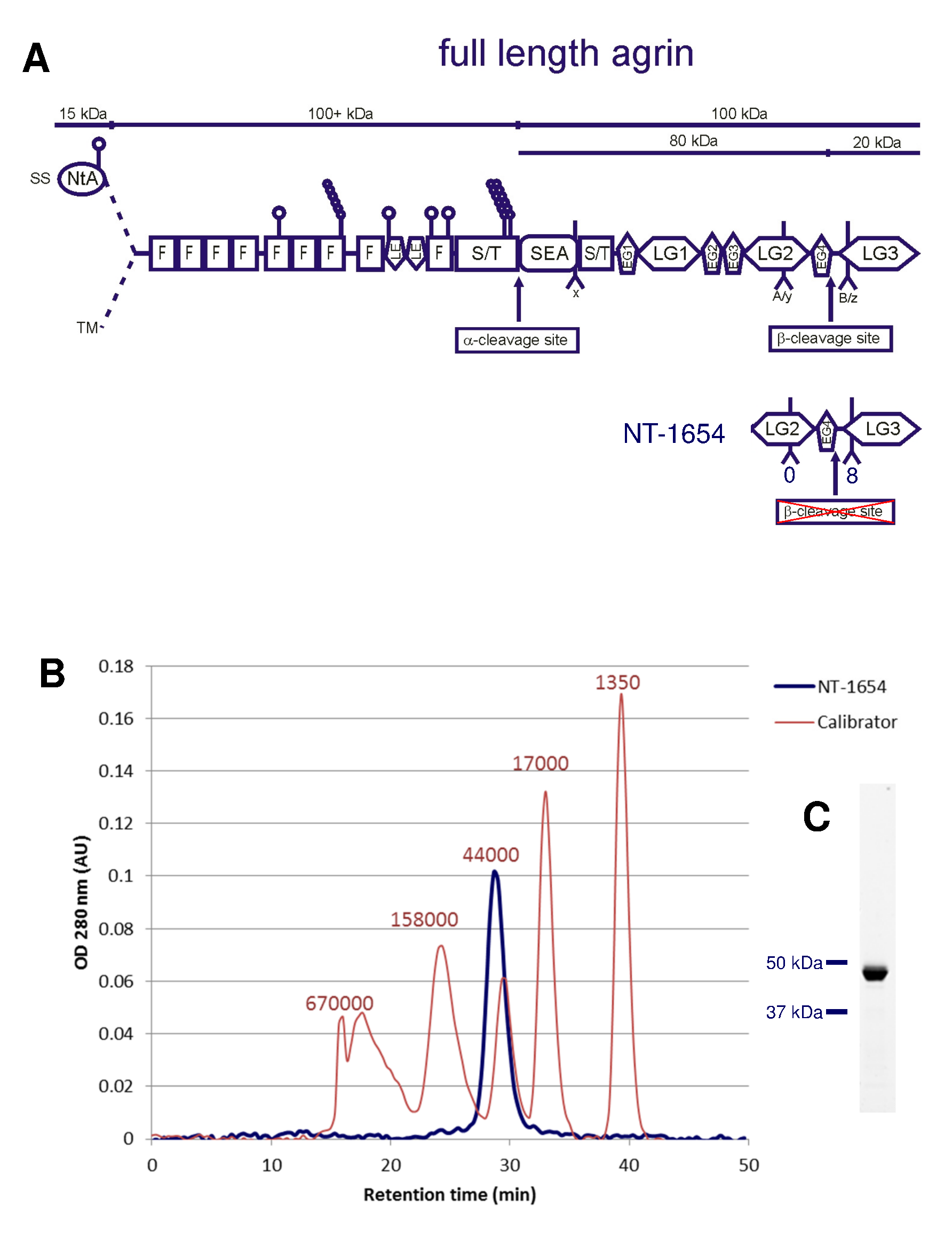

Supplement: Figure S1 — Domain structure and appearance of NT-1654. A: Schematic drawing of full length agrin and NT-1654 (modified from [22]). NT-1654 consists of the LG2, EGF4 and LG3 domains of agrin. At the y-site, no additional amino acids are inserted while at the z-site 8 amino acids are inserted. The neurotrypsin β-cleavage site is mutated at the P1 residue by replacing the lysine by alanine. Neurotrypsin cleavage sites are indicated as α-cleavage site and β-cleavage site. Heparan sulphate glycan chain attachment sites are marked by lollipop chains, glycosylation sites by lollipops. The three splice sites x, A/y, B/z are indicated. NtA: N-terminal agrin domain; F: follistation domains; LE: laminin EGF-like domains; S/T: serine/threonine rich segments; SEA: sea urchin sperm protein, enterokinase, and agrin domain; EG: EGF-like domain; LG: laminin globular like domain; SS: signal sequence; TM: transmembrane segment. B: Size exclusion chromatography revealed that NT-1654 is monomeric. It elutes at the expected retention time. The elution profile of the Biorad 151-1901 gel filtration standard is depicted in red and the corresponding molecular weights of the standard proteins are indicated above the curve. C: The protein could be purified to a high degree of purity as judged by Sypro ruby staining on an SDS-PAGE gel. 2 ug of protein were loaded. (TIF) [file pone.0088739.s001.tif]

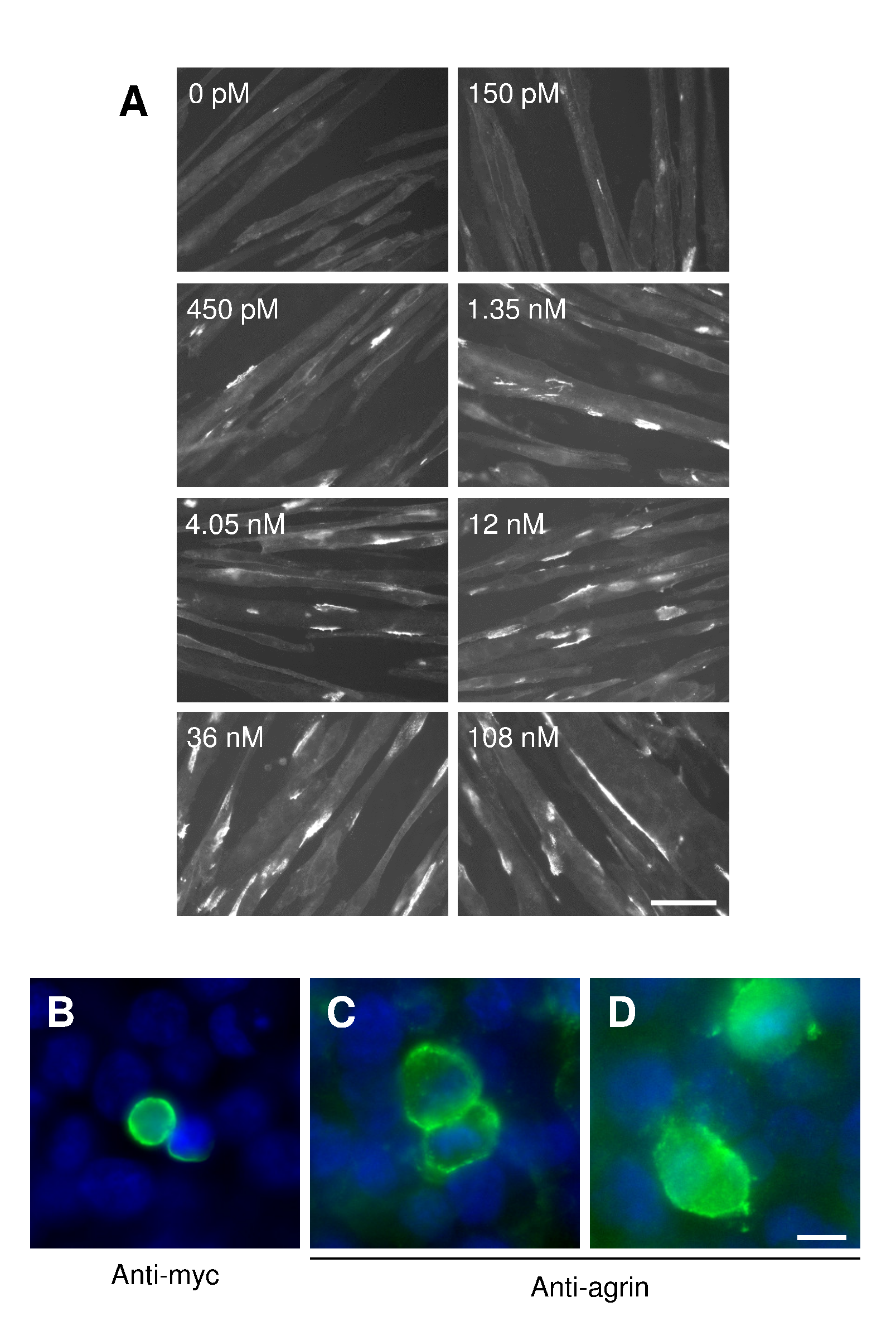

Supplement: Figure S2 — Related to Figure 1: NT-1654 has AChR clustering activity and directly binds to Lrp4. A: NT-1654 induces ectopic AChR clustering in vitro, related to Fig. 1A. C2C12 myotubes were subjected to various amounts of NT-1654 as indicated and stained with α-bungarotoxin to visualize clusters of acetylcholine receptors (AChRs). The number and area of AChR clusters were dose-dependent. The cluster area (percentage of myotube area) was then used to determine the EC50 (see Fig. 1A). Scale bar: 50 µm. B: HEK293 cells were transfected with Lrp4-myc and positive cells were detected by anti-myc antibodies (green). Cell nuclei were visualized by DAPI staining. C: HEK 293 cells were transfected with Lrp4-myc and NT-1639 (a human version of NT-1654) was added. NT-1639 that was bound to Lrp4 was detected by the 204 anti-agrin antibody (green). Cell nuclei were visualized by DAPI staining. D: As a positive Control, a C21 fragment of human agrin is shown to bind to Lrp4 (green). Scale bar: 20 µm. (TIF) [file pone.0088739.s002.tif]

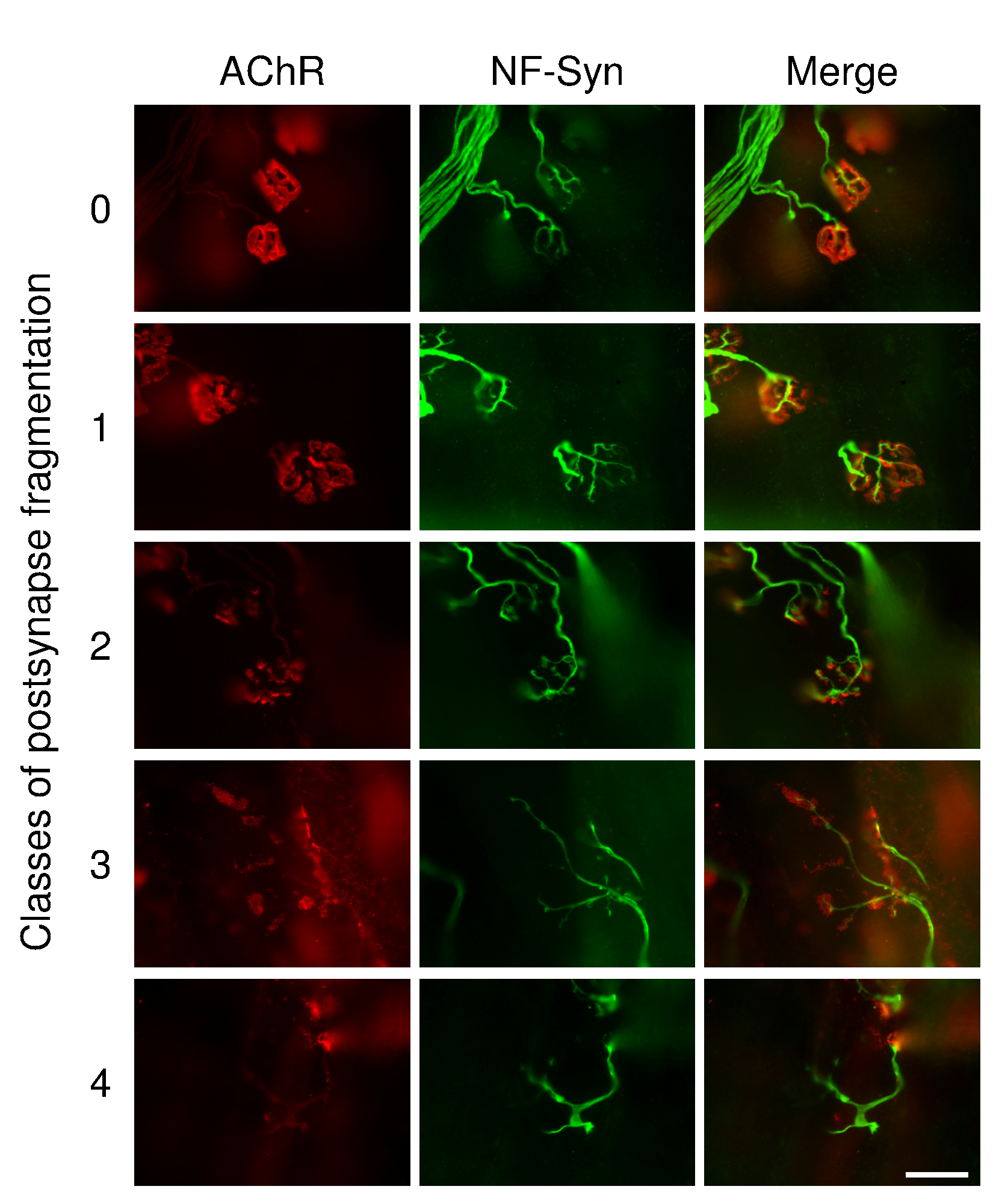

Supplement: Figure S3 — Related to Figure 3: Categorization of postsynapse fragmentation. In order to quantify the severity of postsynapse fragmentation, we observed the NMJs under the Leica DM 5000B fluorescence microscope with 63x objective. The postsynaptic AChRs were stained with Alexa-555 conjugate α-bungarotoxin (red) and the presynapse was stained with anti-neurofilament and synaptophysin antibodies (NF-Syn; green). Class 0: normal NMJ without fragmentation; class 1: light fragmentation with pretzel like postsynapse; class 2: intermediate fragmentation; class 3: severe fragmentation, the pretzel like postsynapse could not be recognized; class 4: the postsynapse largely or completely disappeared. Scale bar: 50 µm. (TIF) [file pone.0088739.s003.tif]

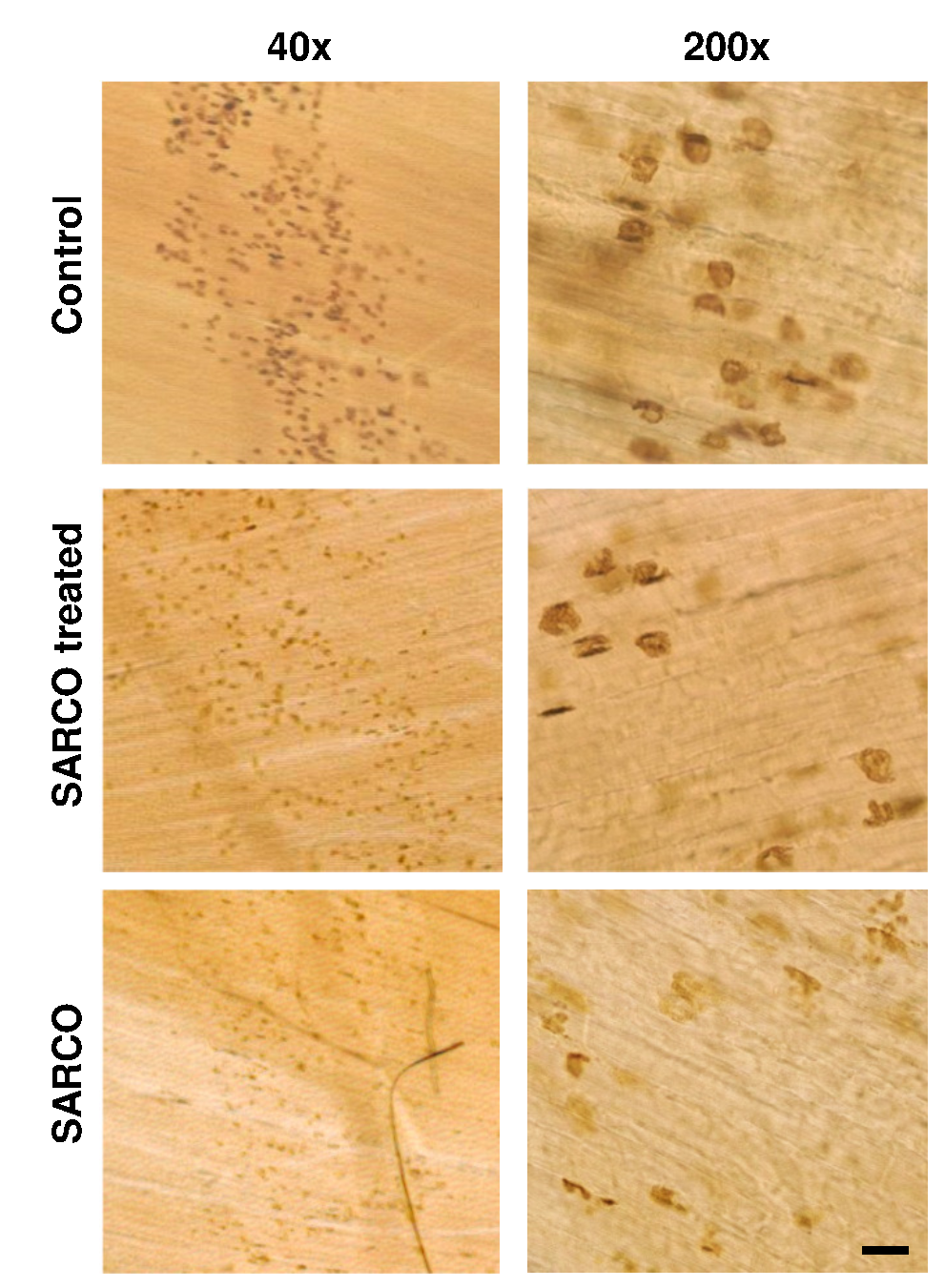

Supplement: Figure S4 — Acetycholine esterase activity on diaphragm of NT-1654 and vehicle treated SARCO mice. Koelle-staining of diaphragm of P30 mice as indicated. Left panels: 40 x, right panels 200 x. The Koelle-staining visualizes the acetylcholine esterase activity in the NMJs. NMJs of Control mice are stained intense and are arranged in a distinct narrow endplate band. The shape of the NMJs is roundish and some perforations can be observed. NMJs of SARCO mice are faintly stained and fragmented. The endplate band is broader as in Controls, comparable to that in treated SARCO mice. Staining in treated SARCO mice is less intense but distinct. The shape of the NMJs is mostly roundish with some perforations at the edge. Scale bar given for 200 x magnitude: 50 µm. (TIF) [file pone.0088739.s004.tif]
